# Protective effect of melatonin on intestinal mucosal injury in intermittent hypoxic rats

**DOI:** 10.3389/fphar.2026.1982966

**Published:** 2026-10-29

**Authors:** QianCheng Chen, Ziyang Zeng, JiaFeng Luo, XinHao Yuan, AiJing Guo, Jiabin Zhang, Aishuang Fu, YanLei Ge

**Affiliations:** 1 Department of Respiratory and Critical Care Medicine, North China University of Science and Technology Affiliated Hospital, Tangshan, Hebei, China; 2 North China University of Science and Technology School of Clinical Medicine, Tangshan, Hebei, China

**Keywords:** intermittent hypoxia, intestinal mucosal injury, intestinal villi, melatonin, oxidative stress

## Abstract

**Objective:**

To investigate the effects of melatonin (MT) on intestinal mucosal injury associated with intermittent hypoxic (HI) in rats.

**Methods:**

Thirty-six male Wistar rats were randomly divided into control (Ctrl), HI, MT groups (n = 12 per group). Each group was further divided into 2- and 4-week subgroups (n = 6 per group per time point), with different animals used at the two terminal sampling time points. Rats in the Ctrl group were exposed to compressed air, whereas rats in the HI and MT groups underwent intermittent hypoxia for 7 h daily. MT (20 mg/kg) was administered intraperitoneally before each daily exposure, while rats in the Ctrl and HI groups received an equivalent volume of vehicle. At the designated 2- or 4-week endpoint, the sampling procedure was initiated immediately after completion of the final daily exposure. Small intestinal tissue and central venous blood were collected. Intestinal histopathological changes were assessed by hematoxylin and eosin staining and Chiu scoring, and villus area, height, thickness, and crypt depth were measured. Serum ROS, MDA, SOD, and DAO levels were determined.

**Result:**

Intermittent hypoxia was associated with intestinal histopathological injury and significant reductions in villus area, villus height, villus thickness, and crypt depth at both 2 and 4 weeks. At 2 weeks, no significant differences in villus morphometric parameters were observed between the MT and HI groups, at 4 weeks, villus area and height were significantly greater in the MT group than in the HI group. At 2 weeks, SOD activity was significantly higher in the MT group than in the HI group, whereas ROS, MDA, and DAO levels did not differ significantly between these groups. At 4 weeks, the MT group showed significantly lower ROS, MDA, and DAO levels and significantly higher SOD activity than the HI group. Histological findings also indicated less severe intestinal mucosal injury in MT-treated rats.

**Conclusion:**

Intermittent hypoxia was associated with intestinal mucosal injury and changes in oxidative stress-related biochemical indicators in rats. Melatonin treatment was associated with attenuation of histopathological injury and a more favorable biochemical profile, with these differences being more evident at 4 weeks.

## Introduction

Obstructive sleep apnea syndrome (OSAHS) has been shown to cause an increase in a variety of inflammatory mediators in the body. It has been recognized as one of the risk factors for diabetes mellitus, hypertension, and cardiovascular and cerebral vascular diseases due to the long-term chronic hypoxia caused by OSAHS (Durgan et al., 2016). Some studies at home and abroad have shown that long-term chronic inflammatory stimulation can lead to the disruption of microbial homeostasis in the intestinal tract, which in turn leads to the development of intestinal-borne endotoxemia, and can exacerbate the severity of the body’s oxidative stress (Hansen et al., 2015; Pussinen et al., 2011). Melatonin (MT), as an endocrine hormone secreted by the pineal gland, regulates physiological activity processes in addition to the sleep cycle, pubertal development, and seasonal acclimatization, and MT and its metabolites have been found to have important roles such as antioxidant and inhibition of inflammatory responses (Carpentieri et al., 2012). In this experiment, intermittent hypoxia rats were used as a model to explore the protective effect of melatonin on intestinal mucosal injury in intermittent hypoxia rats, which will provide a theoretical basis for future clinical studies.

## Materials and methods

### Animals, experimental design, and grouping

The intermittent hypoxia experimental system consisted mainly of a programmable control system, a gas supply system providing high-purity nitrogen (>95%), and a hypoxia chamber. The oxygen concentration inside the hypoxia chamber was continuously monitored in real time throughout the exposure period to verify that the predefined hypoxia–reoxygenation conditions were achieved. The gas supply concentration and flow rate were adjusted accordingly to maintain the target oxygen concentration. Thirty-six 6-week-old male Wistar rats, weighing 170 ± 10 g, were obtained from the Experimental Animal Center of North China University of Science and Technology (Laboratory Animal Use License No. SYXK [Hebei] 2015-0038). All animals were housed under standard laboratory conditions and allowed to acclimatize for 1 week before the formal experiment. The study protocol was approved by the Institutional Animal Ethics Committee (Ethical Review No. LX2019069). After acclimatization, the 36 rats were randomly assigned using a random-number method to three groups: the control (Ctrl) group (*n* = 12), intermittent hypoxia (HI) group (*n* = 12), and melatonin (MT) group (*n* = 12). Each group was further divided into two subgroups according to the sampling time point. Six rats from each group were sacrificed and sampled after 2 weeks of intervention, while the remaining six rats were sacrificed and sampled after 4 weeks of intervention. Thus, the sample size was *n* = 6 per group at each time point, and different animals were used for the 2-week and 4-week assessments. The overall experimental design and timeline are shown in Figure 1.

**FIGURE 1:**
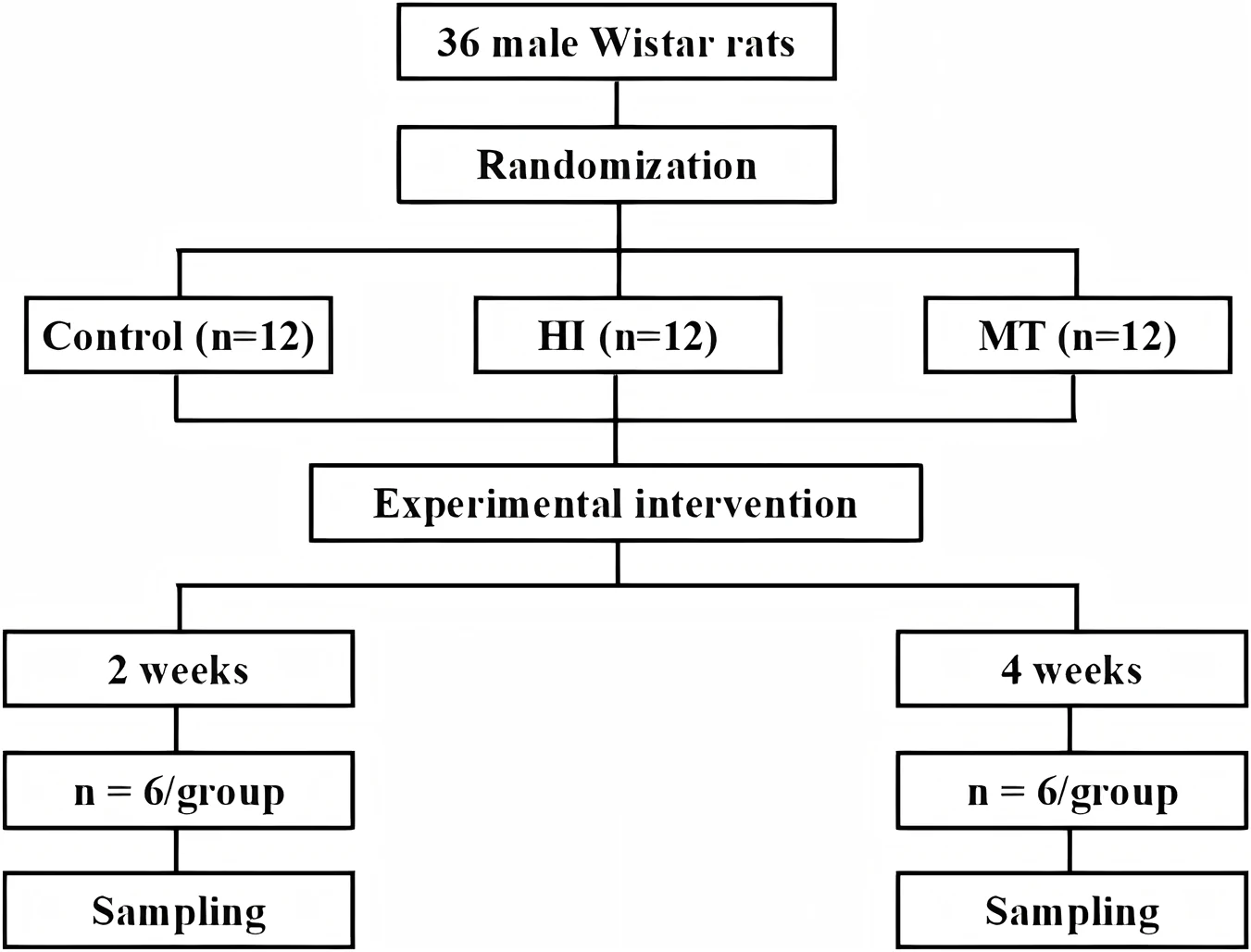
Experimental design and timeline.

### Intermittent hypoxia and melatonin treatment

During the experimental period, in accordance with the physiological activity pattern of rodents, all experimental rats were placed in the hypoxia chamber from 9:00 to 16:00. In the Ctrl group, compressed air was continuously injected into the chamber at a flow rate of 10 L/min, maintaining the oxygen concentration at 21% ± 0.5%. In the MT group, MT (ALEXIS, Switzerland) was dissolved in anhydrous ethanol and subsequently diluted with saline to achieve a final ethanol concentration of <2%. MT was administered intraperitoneally at 20 mg/kg before the start of each daily experiment. To control for the potential effects of the injection procedure and vehicle, rats in the Ctrl and HI groups received an equivalent volume of the same ethanol/saline vehicle (without melatonin) via intraperitoneal injection according to the same daily administration schedule as the MT group. The rats in the HI and MT groups were then subjected to the same intermittent hypoxia treatment, consisting of three stages: hypoxic exposure with high-purity nitrogen (30 s, 10 L/min), rapid reoxygenation with compressed air (40 s, 10 L/min), and slow reoxygenation with compressed air (50 s, 5 L/min), resulting in a total cycle duration of 120 s. During the hypoxic phase, the oxygen concentration in the chamber was reduced to 5% ± 0.5%, followed by reoxygenation to approximately 21%. After the completion of the daily experimental procedures, all rats were returned to the same housing environment and provided with the same diet.

### Anaesthesia, euthanasia, and sample collection

At the designated sampling time points (2 or 4 weeks after the start of the intervention), blood and intestinal tissue samples were collected immediately after completion of the final daily intermittent hypoxia exposure. For the Ctrl group, sample collection was performed immediately after completion of the corresponding daily chamber exposure to ensure comparable sampling conditions among groups. rats were anesthetized via intraperitoneal injection of 20% urethane at a dose of 5 mL/100 g body weight. Adequate anesthesia was confirmed by the absence of corneal reflex and pedal withdrawal reflex. Animals were maintained under deep anesthesia throughout the surgical procedures. Following confirmation of a surgical plane of anesthesia, rats were euthanized by exsanguination via central venous blood collection. Death was confirmed by cessation of heartbeat and respiratory movement. All procedures were conducted in accordance with institutional guidelines for the care and use of laboratory animals and complied with relevant ethical regulations for animal experimentation. Immediately after euthanasia, small intestinal tissues approximately 2 cm proximal to the ileocecal valve were harvested under sterile conditions for further histological and biochemical analyses.

### Histological and morphometric assessment

Small intestinal tissue obtained approximately 2 cm proximal to the ileocecal valve was immediately fixed in 10% neutral buffered formalin, dehydrated through a graded ethanol series, embedded in paraffin, serially sectioned, stained with hematoxylin and eosin (HE), mounted, and examined under a light microscope. Histological and morphometric assessments were performed in all six animals from each group at each time point (n = 6 per group per time point). Histological injury was evaluated using the Chiu 6-point scoring system. For Chiu scoring, five well-oriented microscopic fields were evaluated for each animal, and the scores from the five fields were averaged to obtain a single representative score for each animal. For morphometric analysis, five well-oriented intestinal villi were selected from each animal, and villus area, villus height, villus thickness, and crypt depth were measured. The measurements from the five villi were averaged to obtain a single representative value for each animal before statistical analysis. Both Chiu histological scoring and morphometric measurements were performed by an investigator blinded to group assignment. Morphometric measurements were performed using Image-Pro Plus version 6.0.

### Biochemical measurements

Serum DAO was assessed as a biochemical indicator associated with intestinal mucosal injury (Huang et al., 2008). Serum levels of malondialdehyde (MDA) (Wuhan Cloud-Clone Corp., Wuhan, China; Cat. No. SEJ298Hu), reactive oxygen species (ROS) (Wuhan Cloud-Clone Corp., Wuhan, China; Cat. No. SEB971Ra), superoxide dismutase (SOD) (Wuhan Cloud-Clone Corp., Wuhan, China; Cat. No. SES134Ca), and diamine oxidase (DAO) (Wuhan Cloud-Clone Corp., Wuhan, China; Cat. No. HEA597Ge) were measured in central venous blood using the corresponding assay kits according to the manufacturers’ instructions.

### Statistical analysis

Data are presented as *mean* ± *SD*. Statistical analyses were performed using SPSS version 22.0 and GraphPad Prism. The effects of treatment (Ctrl, HI, and MT), exposure duration (2 and 4 weeks), and the treatment × time interaction were analyzed using two-way ANOVA. When appropriate, Tukey’s multiple comparisons test was performed to evaluate simple effects between treatment groups within each time point, with adjustment for multiple comparisons. Exact adjusted P values are reported where appropriate, and a two-sided *P* value <0.05 was considered statistically significant.

## Results

### Histopathological changes in the small intestine

Representative HE-stained sections of the small intestine are shown in Figure 2. In both the 2-week and 4-week Ctrl groups (① and ②, respectively), the intestinal mucosal architecture was generally preserved, with relatively intact epithelium and well-organized villi. In the 2-week HI group (③), the intestinal villi appeared shortened, with partial disruption of the villous structure and mild mucosal congestion. More pronounced histopathological alterations were observed in the 4-week HI group (④), including disruption of villous architecture, epithelial damage, and mucosal congestion and edema. In the 2-week MT group (⑤), histological alterations, including partial villous disruption and mild mucosal congestion and edema, were still observed. In the 4-week MT group (⑥), some villous structural abnormalities and mucosal edema remained; however, the histological alterations appeared less pronounced than those observed in the corresponding 4-week HI group. These qualitative histological observations were further evaluated using Chiu histological scoring and quantitative villus morphometry.

**FIGURE 2:**
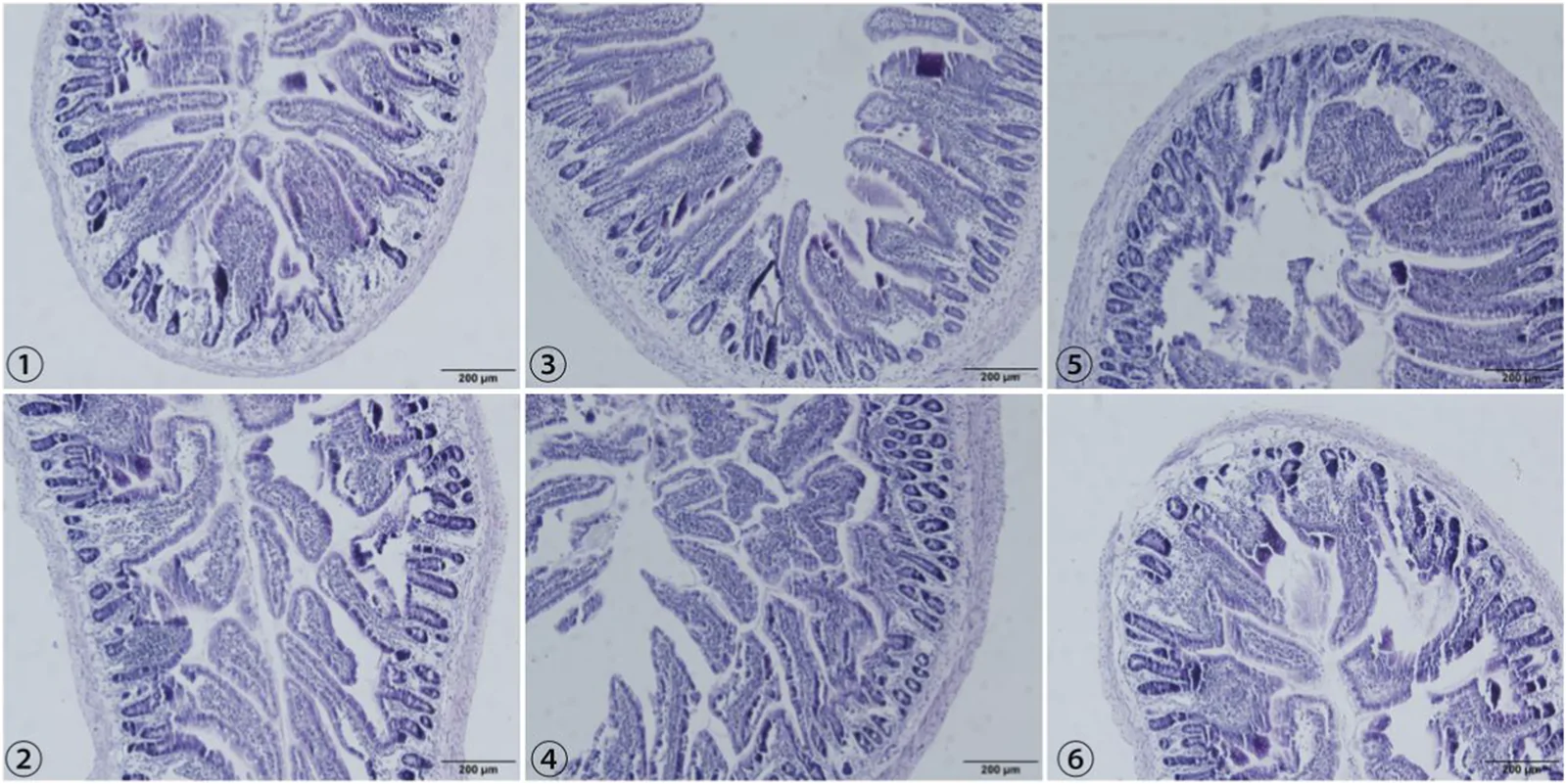
Representative hematoxylin and eosin (HE)-stained sections of small intestinal tissue from each experimental group. ① 2-week Ctrl group; ② 4-week Ctrl group; ③ 2-week HI group; ④ 4-week HI group; ⑤ 2-week MT group; and ⑥ 4-week MT group. Original magnification, ×100; scale bar = 200 μm.

Assessment of small intestinal villus morphology and damage: According to Chiu’s 6-point scoring system (Chiu et al., 1970), five intestinal villus morphologies were selected from each group of rats for scoring. The scores for the 2-week Ctrl group, 4-week Ctrl group, 2-week HI group, 4-week HI group, 2-week MT group, and 4-week MT group were as follows: 0; 0 (3.05 ± 0.46) (4.15 ± 0.53) (2.88 ± 0.30) (2.40 ± 0.48). The 4-week HI group had the highest score, indicating that intestinal villus damage gradually worsened with prolonged intermittent hypoxia. The MT group had lower scores than the HI group (*P* < 0.05), suggesting that the extent of intestinal villus damage was reduced compared to the same-period HI group.

Image processing software Image-Pro Plus Version 6.0 was used to process small intestine tissue images. Five intestinal villi were measured consecutively for each specimen, with results expressed as relative values (Table 1). At both 2 and 4 weeks, the HI groups showed significantly reduced villus area, villus height, villus thickness, and crypt depth compared with the corresponding Ctrl groups (*P* < 0.05). At 2 weeks, although the MT group also showed lower values for all four morphological parameters than the Ctrl group (*P* < 0.05), no significant differences were observed between the 2-week MT and 2-week HI groups (*P* > 0.05). At 4 weeks, the MT group showed significantly greater villus area and villus height than the 4-week HI group (*P* < 0.001), whereas the differences in villus thickness and crypt depth between the two groups were not statistically significant (*P* > 0.05). Compared with the 4-week Ctrl gro*up, villus thickness remained significantly reduced in the 4-week MT group (P < 0.01), while villus area, villus height, and crypt depth showed no significant differences (P* > 0.05). These findings indicate that intermittent hypoxia was associated with impaired intestinal villus morphology, while MT treatment partially ameliorated these alterations, particularly in villus area and height after 4 weeks of treatment.

**TABLE 1:** Comparison of intestinal villus area, height, thickness, and crypt depth among groups of rats.

Group/Project	Intestinal villus area	Intestinal villus height	Intestinal villus thickness	Crypt depth
2-week ctrl group	248,743.79 ± 29,017.76	1317.20 ± 165.85	1712.99 ± 240.17	543.52 ± 127.45
4-week ctrl group	266,618.90 ± 39,889.57	1323.89 ± 158.35	1776.65 ± 259.35	514.74 ± 130.68
2-week HI group	205,375.65 ± 10,677.99^*^	955.79 ± 121.41^***^	1161.75 ± 146.51^***^	340 ± 43.95^**^
4-week HI group	155,929.90 ± 23,805.46^####^	774.17 ± 73.13^####^	1032.57 ± 128.59^####^	318.87 ± 76.30^##^
2-week MT group	204,747.35 ± 18,036.99^*^	1066.50 ± 123.95^*^	1212.98 ± 211.70^***^	393.16 ± 105.82^*^
4-week MT group	231,069.31 ± 30,622.49^&&&^	1167.17 ± 154.92^&&&&^	1321.22 ± 212.51^##^	429.85 ± 48.19

Data are presented as *mean* ± *SD* (*n* = 6). Statistical analysis was performed using two-way ANOVA, followed by Tukey’s multiple-comparisons test for simple effects within each time point.

^*^ *P* < 0.05.

^**^ *P* < 0.01, and ^***^ *P* < 0.001 vs. the 2-week Ctrl group.

^##^ *P* < 0.01 and.

^####^ *P* < 0.0001 vs. the 4-week Ctrl group.

^&&&^ *P* < 0.001 and.

^&&&&^ *P* < 0.0001 vs. the 4-week HI, group.

### Comparison of serum biochemical indicator levels among groups of rats

Comparison of serum biochemical indicators among the experimental groups revealed significant differences (Table 2). At 2 weeks, serum DAO levels were significantly higher and SOD levels were significantly lower in the HI group than in the Ctrl group (P < 0.001 and P < 0.05, respectively), whereas no significant differences in ROS or MDA levels were observed between the two groups (*P* > 0.05). The 2-week MT group showed significantly higher SOD activity than the 2-week HI group (*P* < 0.0001), while ROS, MDA, and DAO levels did not differ significantly between the two groups (*P* > 0.05). At 4 weeks, DAO levels were significantly elevated in the HI group compared with the Ctrl group (*P* < 0.0001), whereas the differences in ROS, MDA, and SOD levels between the two groups were not statistically significant (*P* > 0.05). Compared with the 4-week HI group, the 4-week MT group showed significantly lower ROS, MDA, and DAO levels (*P* < 0.01, *P* < 0.05, and *P* < 0.01, respectively) and significantly higher SOD activity (*P* < 0.01). These findings suggest that MT treatment was associated with a more favorable serum biochemical profile after 4 weeks of intermittent hypoxia, characterized by reduced ROS, MDA, and DAO levels and enhanced SOD activity.

**TABLE 2:** Comparison of serum ROS, MDA, SOD, and DAO levels among groups of rats.

Group/Project	ROS(IU/ml)	MDA(nmol/ml)	SOD(U/ml)	DAO(IU/ml)
2-week ctrl group	444.15 ± 34.22	2.81 ± 0.54	15.88 ± 1.15	11.96 ± 2.03
4-week ctrl group	466.71 ± 18.31	2.70 ± 0.40	15.41 ± 0.75	12.89 ± 2.30
2-week HI group	519.44 ± 105.08	2.81 ± 0.70	14.29 ± 0.57^*^	20.63 ± 4.32^***^
4-week HI group	551.34 ± 102.34	3.53 ± 0.15	16.36 ± 1.28	24.16 ± 4.57^####^
2-week MT group	504.49 ± 39.03	3.16 ± 0.66	17.43 ± 0.88^*††††^	18.29 ± 4.52^*^
4-week MT group	428.56 ± 19.67^&&^	2.54 ± 0.90^&&^	18.40 ± 0.58^####&&^	16.85.±3.54^&&^

Data are presented as *mean* ± *SD* (*n* = 6). Statistical analysis was performed using two-way ANOVA, followed by Tukey’s multiple-comparisons test for simple effects within each time point.

^*^ *P* < 0.05 and ^***^ *P* < 0.001 vs. the 2-week Ctrl group.

^††††^ *P* < 0.0001 vs. the 2-week HI, group.

^####^ *P* < 0.0001 vs. the 4-week Ctrl group.

^&^ *P* < 0.05 and.

^&&^ *P* < 0.01 vs. the 4-week HI, group.

## Discussion

Under normal physiological conditions, a substantial proportion of cardiac output is distributed to the gastrointestinal tract. However, during physiological stress, redistribution of the circulation may reduce gastrointestinal blood flow and increase the susceptibility of the intestinal mucosa to injury. The repeated hypoxia–reoxygenation episodes characteristic of OSAHS may induce pathophysiological changes resembling aspects of ischemia–reperfusion injury and may contribute to oxidative stress (Lim et al., 2009). Previous animal studies have also reported that hypoxic exposure can induce pathological alterations in the intestinal epithelium and villi. In the present study, intermittent hypoxia was associated with marked histopathological and morphometric alterations in the small intestine. Compared with the corresponding Ctrl groups, the HI groups showed significant reductions in villus area, villus height, villus thickness, and crypt depth at both 2 and 4 weeks. Histological examination also revealed structural abnormalities of the intestinal villi, together with mucosal edema and congestion. In addition, serum DAO levels, assessed as a biochemical indicator associated with intestinal mucosal injury, were significantly higher in the HI group than in the corresponding Ctrl group at both 2 and 4 weeks. Taken together, these histological, morphometric, and biochemical findings support the presence of intestinal mucosal injury following intermittent hypoxia. However, intestinal permeability was not directly measured in the present study. Therefore, these findings should not be interpreted as direct evidence of increased intestinal permeability or impaired barrier function (Pussinen et al., 2011; Hartstra et al., 2015).

OSAHS is a sleep-related breathing disorder associated with multiple systemic complications, and its potential effects on gastrointestinal homeostasis have received increasing attention. Understanding intestinal injury associated with intermittent hypoxia may therefore provide further insight into the gastrointestinal consequences of OSAHS. Nevertheless, because the present study used an experimental rat model and did not directly assess intestinal permeability or clinical gastrointestinal outcomes, the clinical implications of these findings should be interpreted cautiously. Further studies are required to determine the relationship between intermittent hypoxia, intestinal mucosal injury, and intestinal barrier function in clinically relevant settings.

MT is an endogenous molecule with well-established antioxidant and free-radical-scavenging properties (Hardeland et al., 2015). In the present study, MT treatment was associated with attenuation of several intestinal histopathological and morphometric alterations induced by intermittent hypoxia. At 2 weeks, however, the morphometric parameters did not differ significantly between the MT and HI groups, despite evidence of less severe histological injury. At 4 weeks, villus area and height were significantly greater in the MT group than in the HI group, whereas villus thickness and crypt depth did not differ significantly between these groups. These findings suggest that the association between MT treatment and preservation of intestinal morphology was more evident for some morphometric parameters after 4 weeks of exposure, rather than demonstrating a uniform effect across all parameters and time points. The serum biochemical findings also differed between the two assessment time points, although they do not by themselves establish a time-dependent treatment effect. At 2 weeks, SOD activity was significantly higher in the MT group than in the HI group, whereas ROS, MDA, and DAO levels did not differ significantly between these groups. At 4 weeks, the MT group showed significantly lower ROS, MDA, and DAO levels and significantly higher SOD activity than the HI group. Thus, the biochemical changes associated with MT treatment were more evident at 4 weeks. Together with the histological findings, these results suggest that MT treatment was associated with attenuation of intestinal mucosal injury and changes in oxidative stress-related biochemical indicators during intermittent hypoxia.

The antioxidant properties of MT reported in previous studies may provide a possible explanation for these observations. SOD represents an important component of endogenous antioxidant defense, whereas ROS and MDA are commonly associated with oxidative stress and lipid peroxidation. Previous studies have suggested that MT can directly scavenge reactive species and modulate antioxidant defense systems (Hardeland et al., 2015; Yoo et al., 2016). Other studies have also proposed that MT may influence nitric oxide-related pathways, inflammatory responses, mitochondrial function, and apoptosis (Sarti et al., 2013). However, these mechanisms were not directly investigated in the present study. In particular, we did not measure inflammatory cytokines, nitric oxide synthase activity, mitochondrial function, or apoptosis-related markers. Therefore, the present findings cannot establish whether the observed effects associated with MT treatment were mediated through anti-inflammatory, mitochondrial, or anti-apoptotic mechanisms. These mechanisms should be regarded as potential explanations based on previous literature rather than conclusions derived from the present experiments.

Several limitations of the present study should be acknowledged. First, systemic physiological or molecular markers of hypoxia, such as arterial blood gases, oxygen saturation, or HIF-1α expression, were not assessed. Although the oxygen concentration within the hypoxia chamber was continuously monitored in real time to verify the intended hypoxia–reoxygenation exposure, chamber oxygen concentration does not directly quantify the degree of systemic hypoxemia experienced by individual animals. Second, only a single dose of MT (20 mg/kg) was evaluated; therefore, dose–response relationships and the optimal dose of MT could not be determined. Third, only male Wistar rats were included, which may limit the generalizability of the findings across sexes and animal strains. Fourth, although intestinal histopathology and morphometry and serum ROS, MDA, SOD, and DAO were assessed, intestinal tight-junction proteins, such as occludin, ZO-1, and claudins, as well as inflammatory cytokines, mitochondrial function, and apoptosis-related markers, were not evaluated. In addition, intestinal permeability was not directly measured. Therefore, the present study cannot establish the molecular mechanisms underlying the observed effects associated with MT treatment or draw definitive conclusions regarding changes in intestinal permeability or restoration of intestinal barrier function. Fifth, the experimental intermittent hypoxia protocol represents a controlled animal model and may not fully reproduce the severity, duration, and complexity of hypoxia–reoxygenation exposure in patients with OSAHS. Therefore, caution is warranted when extrapolating these findings to the clinical setting. Future studies should incorporate different MT doses, both sexes and additional animal strains, physiological measures of hypoxia, direct assessments of intestinal permeability and tight-junction proteins, and inflammatory, mitochondrial, and apoptosis-related markers to further validate these findings and clarify the underlying mechanisms and their translational relevance.

## Conclusion

In summary, intermittent hypoxia was associated with intestinal mucosal injury in rats, as evidenced by histopathological and morphometric alterations and changes in serum biochemical indicators. Melatonin treatment was associated with attenuation of intestinal histopathological injury and improvement in several oxidative stress-related biochemical indicators. These differences were more evident at 4 weeks, particularly for serum ROS, MDA, SOD, and DAO levels and selected villus morphometric parameters. However, the present study did not directly assess intestinal permeability, inflammatory responses, mitochondrial function, apoptosis, or other molecular mechanisms. Therefore, further studies incorporating direct assessments of intestinal barrier function and relevant molecular pathways are needed to clarify the mechanisms underlying the effects of melatonin during intermittent hypoxia.

## Data Availability

The raw data supporting the conclusions of this article will be made available by the authors, without undue reservation.
